# YK-4-279 Inhibits ERG and ETV1 Mediated Prostate Cancer Cell Invasion

**DOI:** 10.1371/journal.pone.0019343

**Published:** 2011-04-29

**Authors:** Said Rahim, Elspeth M. Beauchamp, Yali Kong, Milton L. Brown, Jeffrey A. Toretsky, Aykut Üren

**Affiliations:** Lombardi Comprehensive Cancer Center, Georgetown University Medical Center, Washington, D. C., United States of America; East Carolina University, United States of America

## Abstract

**Background:**

Genomic rearrangements involving the ETS family of transcription factors occur in 40–70% of prostate cancer cases. ERG and ETV1 are the most common ETS members observed in these genetic alterations. The high prevalence of these rearrangements and their biological significance represents a novel therapeutic target for the treatment of prostate cancer.

**Methods and Findings:**

We recently reported the development of YK-4-279, a small molecule inhibitor of EWS-FLI1 oncoprotein in Ewing's Sarcoma. Since ERG and ETV1 belong to the same class of ETS factors as FLI1, we tested the ability of YK-4-279 to inhibit biological functions of ERG and ETV1 proteins in prostate cancer. YK-4-279 inhibited ERG and ETV1 mediated transcriptional activity in a luciferase assay. YK-4-279 also decreased ERG and ETV1 downstream target mRNA and protein expression in *ETV1*-fusion positive LNCaP and *ERG* fusion positive VCaP cells. YK-4-279 reduced the motility of LNCaP cells in a scratch assay and the invasive phenotype of both LNCaP and VCaP cells in a HUVEC invasion assay. Fusion-negative PC3 cells were unresponsive to YK-4-279. SiRNA mediated ERG knockdown in VCaP cells resulted in a loss of drug responsiveness. Concurrently, transient ERG expression in PC-3 cells resulted in increased invasive potential, which was reduced by YK-4-279.

**Conclusion:**

These data demonstrate that YK-4-279 inhibits ERG and ETV1 biological activity in fusion-positive prostate cancer cells leading to decreased motility and invasion. Therefore, YK-4-279 may have an impact on metastasis in prostate cancer and it may be further evaluated for its clinical applications in prostate cancer in addition to Ewing's sarcoma.

## Introduction

Prostate cancer is the most common form of cancer and the second most leading cause of cancer mortality in men. Chromosomal translocations involving the ETS family of transcription factors are present in 40–70% of prostate cancers, including the most clinically aggressive forms [1], [2], [3], [4], [5]. These translocations produce chimeric genes, which fuse the promoter region of an androgen responsive gene, such as *TMPRSS2*, to the coding region of ETS factors, most frequently *ETV1* or *ERG* [6], [7]. These rearrangements lead to androgen dependent regulation of ETS transcription factors and their overexpression. ETS proteins are proto-oncogenes that have been implicated in pathogenesis [8]. They control expression of target genes involved in cell proliferation, apoptosis, invasion and angiogenesis. Over-expression of ETS factors in prostate cancer cells increase cell invasion and induces prostatic intraepithelial neoplasia (PIN) in transgenic mouse models [9]. Depletion of ETS factors *in vitro* reduces motility and invasiveness. ERG and ETV1 depletion also result in reduced tumor growth *in vivo* [7]. Recent results also indicate that *TMPRSS2-ERG* expression is reactivated in castration resistant prostate cancer [10]. Thus, ETS proteins represent a novel target for prevention or treatment of metastatic disease.

We recently reported a small molecule inhibitor of the chimeric protein EWS-FLI1 in Ewing's sarcoma [11]. YK-4-279 inhibits EWS-FLI1 activity, induces apoptosis in Ewing's sarcoma cell lines and slows down tumor growth in mouse xenograft models. FLI1, ERG and ETV1 are Class I ETS factors and share greater than 60% identity and 80% homology in their amino acid sequences [12]. Due to the close homology of FLI1 with ERG and ETV1, we tested the ability of YK-4-279 to inhibit ETS gene activity in prostate cell-lines that demonstrate androgen dependent ERG and ETV1 expression. Our results indicate that YK-4-279 can inhibit ERG and ETV1 dependent transcriptional activity and consequently leads to reduced cell motility and invasion.

## Results and Discussion

### VCaP and LNCaP cells are androgen-responsive and harbor ERG and ETV1 rearrangements

**Figure 1:**
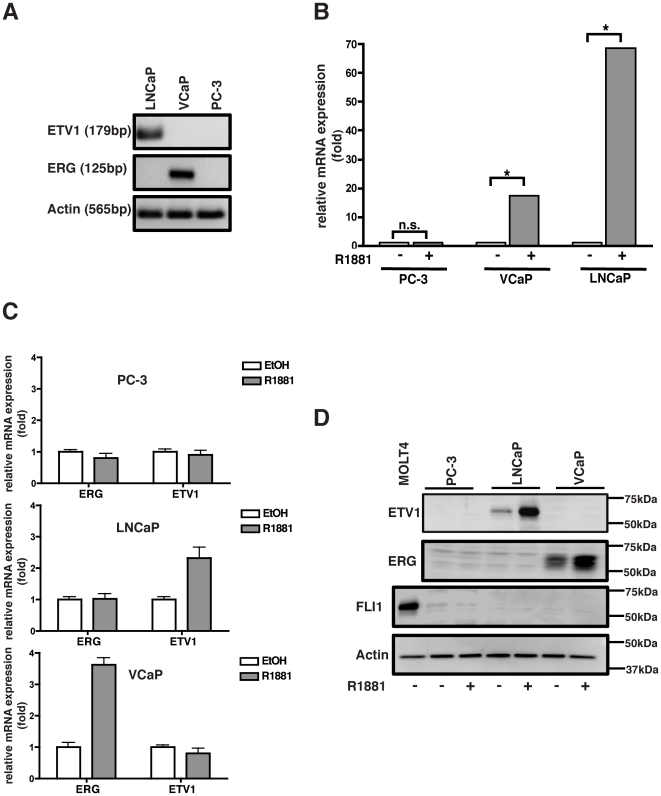
LNCaP and VCaP cells are androgen responsive and harbor *ERG* and *ETV1* rearrangements. a) Prostate cells were analyzed for ETS rearrangement status by performing PCR using rearrangement specific primer. VCaP cells harbor the TMPRSS-ERG rearrangement whereas LNCaP cells contain rearranged ETV1. VCaP and LNCaP cells express ERG and ETV1 protein respectively, under basal conditions. PC-3 cells do not contain either rearrangement and do not express ERG or ETV1. b) VCaP and LNCaP cells express PSA in response to R1881 treatment. PC-3 cells are not androgen responsive. Cells were treated with 10 nM R1881 for 48 hours and PSA expression was analyzed by real-time qPCR. Results were normalized to actin. * ; p<0.0001, n.s.; not-significant. c) R1881 stimulation results in increased ERG and ETV1 mRNA in VCaP and LNCaP cells respectively, but not in PC3 cells. RNA was isolated from androgen stimulated cells and used to perform real-time qPCR. Data was normalized to the level of gene expression in the absence of androgen. d) ERG and ETV1 proteins are expressed in VCaP and LNCaP cells respectively, but not in PC-3 cells. Androgen stimulation resulted in increased ERG and ETV1 protein in VCaP and LNCaP cells. Prostate cells did not express FLI1 protein under basal or androgen stimulated conditions. MOLT4 was used as a positive control cell-line for FLI1 expression.

Prostate requires androgens to function properly and androgen responsiveness can be used as a basis for grouping prostate cancer cell lines into either of two categories: androgen-sensitive and androgen-resistant. In the majority of ETS rearrangement cases, the ETS gene is placed under direct regulation of an androgen-responsive gene promoter. In these cases, androgen mediates over-expression of the oncogenic ETS factor. In order to study the effect of ETS inhibitors in prostate cancer, we selected to work with VCaP and LNCaP cell-lines. The VCaP cell-line harbors a TMPRSS2-ERG rearrangement, which occurs via interstitial deletion of the 3 Mb region between TMPRSS2 and ERG on chromosome 21 (Fig. 1a) [6]. The LNCaP cell-line contains a genetic translocation where the entire ETV1 locus is inserted in the last intron of the prostate-specific MIPOL1 region on chromosome 14 (Fig. 1a) [7]. The ERG and ETV1 rearrangements are mutually exclusive to VCaP and LNCaP cells respectively, and are not present in the PC-3 cell line. Thus, the PC-3 cell-line was selected as a negative control for our studies. We validated that both VCaP and LNCaP cells are androgen-sensitive, as demonstrated by an increase in prostate specific antigen (PSA) expression upon stimulation by the synthetic androgen analogue R1881 (Fig. 1b). VCaP and LNCaP cells express ERG and ETV1 proteins under basal conditions owing to the presence of *ETS* rearrangements in these cells. Androgen treatment of these cell-lines, but not PC-3, results in increased ERG and ETV1 mRNA and protein (Fig. 1c and d). These results establish that both VCaP and LNCaP cells are androgen responsive while PC-3 cells are not. Androgen responsiveness of VCaP and LNCaP cells translates to enhanced ETV1 and ERG expression due to prostate cancer specific chromosomal rearrangements.

### YK-4-279 inhibits ERG and ETV1 transcriptional activity

**Figure 2:**
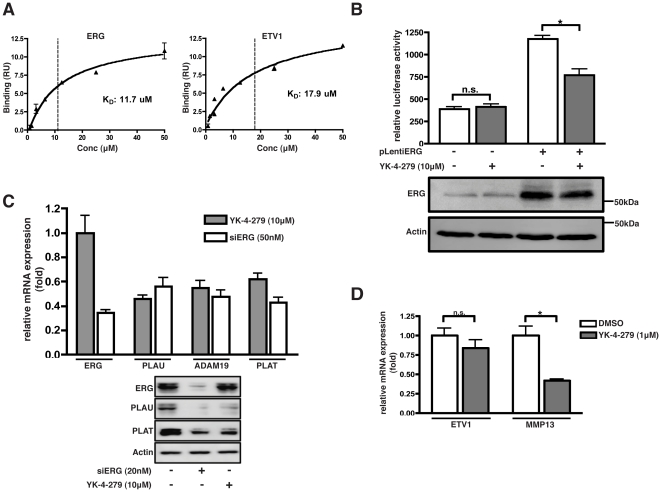
YK-4-279 inhibits ERG and ETV1 transcriptional activity. a) Binding kinetics of YK-4-279 to ERG and ETV1 was determined by surface plasmon resonance. YK-4-279 bound to ERG and ETV1 with a K_D_ of 11.7 µM and 17.9 µM respectively. SPR sensorgrams are provided in supplementary figures (Fig. S1). b) A luciferase assay was performed in Cos-7 cells co-transfected with ERG and an Id-2 reporter luciferase construct. Id-2 promoter activity was decreased upon YK-4-279 treatment without affecting ERG protein levels. * ; p<0.001. c) VCaP cells were treated with 50 nM siERG or 10 µM YK-4-279 for 48 hours and mRNA and protein expression levels of ERG targets were evaluated. YK-4-279 treatment resulted in decreased expression of PLAU, ADAM19 and PLAT mRNA. PLAU and PLAT protein levels were decreased as well. Results were comparable to those obtained by siRNA mediated ERG knockdown. d) LNCaP cells were treated with 1 µM YK-4-279 and ETV1 target gene levels were evaluated. YK-4-279 treatment resulted in decreased gene expression of MMP13 without significant reduction in ETV1 levels. * ; p<0.01.

YK-4-279 targets the EWS-FLI1 oncoprotein in Ewing's Sarcoma [11]. However, the site of interaction with EWS-FLI1 is unknown. Considering the close homology between FLI1, ERG and ETV1, we investigated the effects of YK-4-279 on ERG and ETV1 function. We first evaluated the expression of FLI1 in prostate cells. The human acute lymphoblastic leukemia cell line MOLT4 was used as a control for FLI1 expression, under basal conditions. None of the prostate cells used in this study express FLI1 (Fig. 1d). Hence, the effects of YK-4-279 on prostate cells would not occur as a result of targeting FLI1. Next, we validated the direct interaction between YK-4-279 and recombinant ERG and ETV1 proteins using surface plasmon resonance (SPR). YK-4-279 bound to ERG with an affinity (K_D_) of 11.7 µM and bound to ETV1 with an affinity of 17.4 µM (Fig. 2a, S1). We evaluated YK-4-279 for effects upon ERG transcriptional activity using a transiently transfected 207 bp fragment of the Id2 gene promoter that directs expression of luciferase protein. This minimal Id2 promoter region contains two ETS sites and has been previously shown to bind ERG [13]. Co-transfection of ERG and Id2 reporter resulted in an increase in luciferase activity. Promoter activity was reduced by simultaneous treatment of the cells with YK-4-279, without any appreciable decrease in ERG protein levels (Fig. 2b).

Next, we evaluated the effects of YK-4-279 on expression of endogenous ERG and ETV1 target genes in VCaP and LNCaP cell-lines. We focused on several members of the plasminogen activator pathway such as PLAU, PLAT, MMP13 and ADAM19. These genes mediate an invasive phenotype in several cancers and have been reported as direct targets of ETS transcription factors [9], [14], [15]. Exposure of VCaP cells to 10 µM YK-4-279 for 48 hours resulted in significantly reduced mRNA and protein levels of several ERG target genes, such as PLAU, PLAT and ADAM29. The level of down-regulation was comparable to that obtained by siRNA mediated ERG knock-down in VCaP cells (Fig. 2c). Similarly, YK-4-279 resulted in down-regulation of ETV1 target gene MMP-13 in LNCaP cells (Fig. 2d). It should be noted that this inhibition of ERG and ETV1 protein activity was obtained without any significant decrease in ERG or ETV1 protein levels. These results suggest that YK-4-279 is able to inhibit ERG and ETV1 transcriptional activity in prostate cancer cells, leading to decreased expression of genes that are involved in breakdown of extracellular matrix and metastasis.

### YK-4-279 inhibits ETS mediated prostate cancer cell invasion

Previous studies have suggested that *ETS* gene rearrangements mediate invasion in prostate cancer [7], [9]. To address the question whether YK-4-279 is able to inhibit ERG and ETV1 mediated invasion, we utilized an impedance based endothelial cell invasion assay [16]. This technique involves challenging a confluent monolayer of human umbilical vein endothelial cells (HUVECs) with a second layer of metastatic cells that attach to, and invade the HUVEC monolayer. The retraction of endothelial cell junctions and invasion of prostate cancer cells can be monitored in real-time by measuring the decrease in electrical resistance on gold electrodes [17].

**Figure 3:**
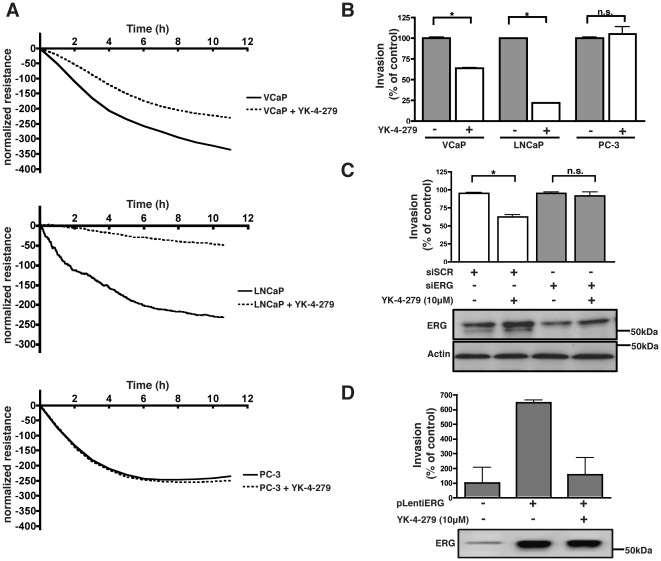
YK-4-279 inhibits ETS mediated prostate cancer cell invasion. a) HUVEC cells forming a confluent monolayer were challenged with LNCaP, VCaP and PC-3 cells with or without YK-4-279. YK-4-279 inhibited VCaP (10 µM) and LNCaP (1 µM) cell invasion of HUVECs, whereas PC-3 cells were not affected. Prostate cells were pre-treated with YK-4-279. Experiments were performed in duplicates and resistance was normalized to the time of addition of invading cells. b) Invasion was quantified at 10 hours post-addition of prostate cancer cells. Results are expressed relative to non-treated conditions. * ; p<0.01. c) ERG expression was reduced in VCaP cells using a C-terminal siRNA probe. ERG knockdown in VCaP cells resulted in a loss of YK-4-279 mediated inhibition of invasion. VCaP cells were pre-treated with 10 µM YK-4-279 for 2 days prior to challenging the HUVEC monolayer. * ; p<0.01, d) Transient ERG expression in PC-3 cells imparted upon the cells a more invasive phenotype. Subsequently, YK-4-279 treatment resulted in decreased invasion. PC-3 cells were treated with YK-4-279 for 24 h prior to challenging the HUVEC monolayer.

A cytotoxicity assay was performed to determine the maximum tolerable dose of YK-4-279 in different prostate cancer cell lines. YK-4-279 did not show any significant reduction in cell growth at 1 µM for LNCaP cells and 10 µM for VCaP and PC3 cells after 2 days of treatment (data not shown). These doses were selected for further functional assays in order to ensure that inhibitory effects of YK-4-279 on invasion and motility, are not as a result of cell death. When HUVEC cells were challenged with LNCaP and VCaP cells, it led to a steep decrease in electric-resistance indicative of cell invasion. Treating these cells with YK-4-279 resulted in significantly decreased invasion of HUVEC cells by LNCaP and VCaP cells. The compound alone had no effect on the HUVEC cell monolayer. YK-4-279 did not inhibit invasion by *ETS*-fusion negative PC-3 cells. (Fig. 3a and b). To ensure that the effects observed were due to inhibition of ETS proteins, we reduced ERG protein expression in VCaP cells using siRNA. This resulted in abrogation of YK-4-279 mediated inhibition of invasion (Fig. 3c). Next, we transiently expressed ERG in PC-3 cells and assayed these cells in the endothelial cell invasion assay. ERG expression alone in PC-3 cells imparted upon these cells a more invasive phenotype. Treatment with YK-4-279 significantly inhibited the ERG mediated increase in invasion (Fig. 3d). Together, these results suggest that YK-4-279 is able to inhibit ETS-mediated invasion of prostate cancer cells, both in cells with endogenous and exogenous high expression of ETS proteins.

### YK-4-279 inhibits ETV1 mediated motility in LNCaP Cells

**Figure 4:**
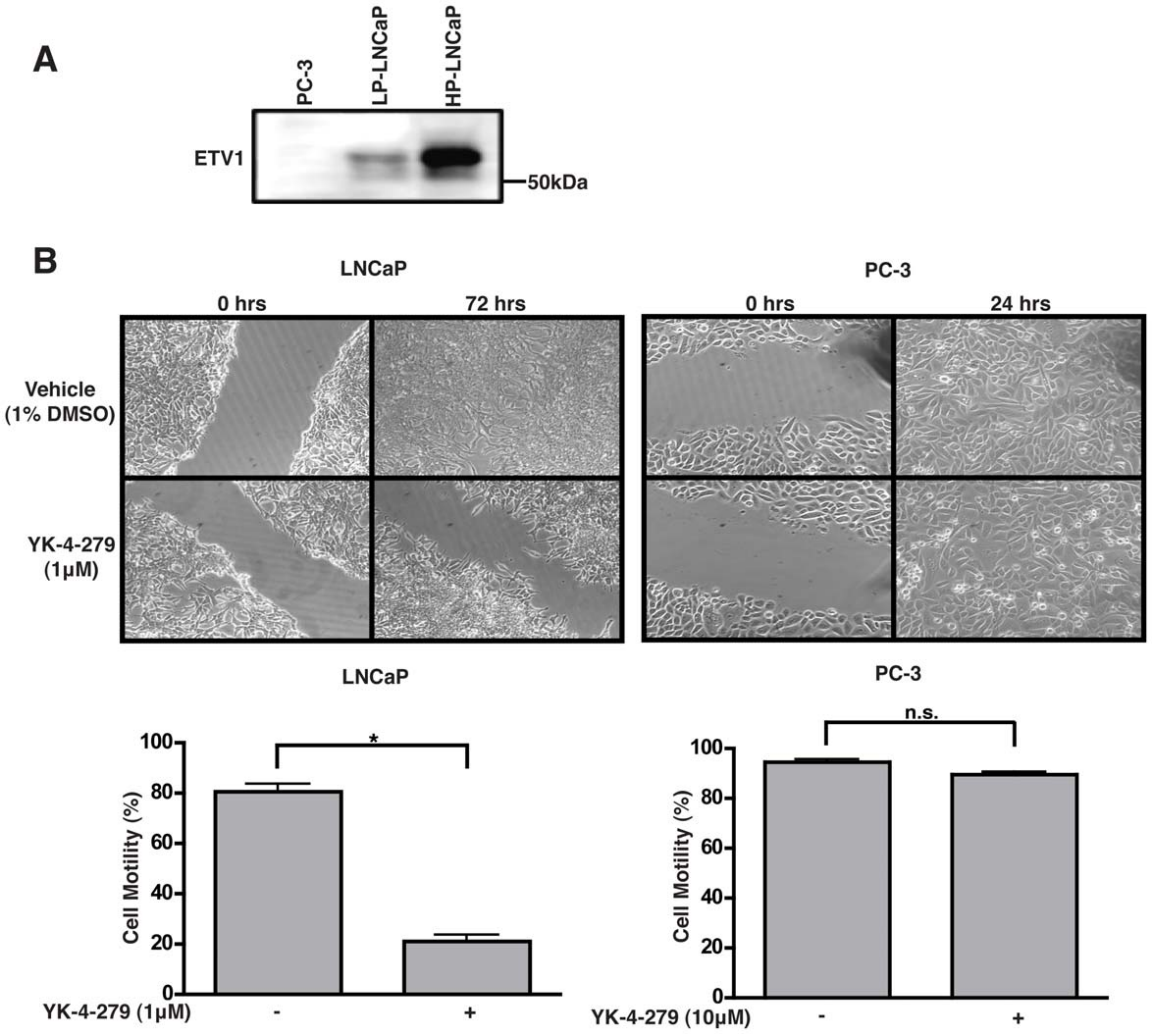
YK-4-279 inhibits ETV1 mediated motility in LNCaP cells. a) High-passage LNCaP cells were analyzed for ETV1 expression levels. HP-LNCaP cells constitutively express higher amounts of ETV1, as compared to PC-3 cells. b) YK-4-279 inhibited motility in a scratch assay in high-passage LNCaP cells, whereas PC-3 cells were unresponsive. Cell motility was quantified by measuring the distance between the migrating cell boundaries. Motility was expressed relative to vehicle treated conditions. * ; p<0.0001.

Next, we tested the effects of YK-4-279 on inhibition of motility of LNCaP cells in a scratch assay. All experiments in previous figures were performed with low passage LNCaP cells (p<30). However, low-passage LNCaP cells were not amenable to this technique as they loosely attach to the cell-culture dish surface. Similarly, VCaP cells grow in clumps and do not form a confluent monolayer. Therefore, we performed scratch assays using high passage LNCaP cells (p>60). High-passage LNCaP cells grow at a much faster rate and are able to form a confluent monolayer [18]. They also express high basal levels of ETV1 (Fig. 4a) [19]. Prior to performing the scratch assay, YK-4-279 was tested for its cytostatic nature and was found to have no effects on cell-proliferation at concentrations used for the scratch assay (Fig. S2). YK-4-279 treatment of LNCaP cells resulted in a significant decrease in cell motility in the scratch assay, while no effects were observed on the motility of the negative control cell-line, PC-3 (Fig. 4b). The scratch assay was also performed with pre-treatment of LNCaP cells with 10 µg/ml mitomycin C for 2 hours prior to scratching the surface. YK-4-279 was able to inhibit LNCaP cell motility in mitomycin treated conditions as well (Fig. S3) These findings suggest that the effects of YK-4-279 on LNCaP cells in scratch assay is not due to cytotoxicity, but solely due to inhibition of cell motility.

The EWS-FLI1 oncoprotein is dependent upon binding to RNA Helicase A (RHA) for its oncogenic function [20]. YK-4-279 induces apoptosis in Ewing's sarcoma cells by blocking the interaction between EWS-FLI1 and RHA. As a possible mechanism for the activity of YK-4-279 in prostate cancer, we tested whether the interaction between an ETS family member and RHA is present in prostate cells as well. While ERG does interact with RHA in prostate cancer cells, YK-4-279 is unable to block this interaction (Fig. S4). We also tested whether YK-4-279 is able to block ERG or ETV1 binding to ETS sites on the DNA by using surface plasmon resonance. YK-4-279 did not inhibit ERG or ETV1 DNA binding (Fig. S5a). Furthermore, chromatin immunoprecipitation was performed to evaluate ERG binding to PLAU promoter in the presence of YK-4-279. Results confirmed Biacore findings that YK-4-279 does not interfere with ERG DNA binding (Fig S5b). It should be noted that Ewing's cells express a truncated FLI1 protein containing only exons 6–9 of FLI1. ETS translocations in prostate cancer, on the other hand, result in the expression of an almost full-length ETS family member. Therefore, we hypothesize that YK-4-279 may inhibit ETV1 and ERG function in prostate cancer cells by preventing protein-protein interactions that are different than EWS-FLI1 partners in Ewing's Sarcoma. Hence, further investigation is required to determine the exact molecular mechanism of YK-4-279 mediated inhibition of ERG and ETV1 function in prostate cancer cells.

The outcome of ETV1 inhibition appears to be more potent than ERG inhibition, in terms of cell-motility and invasion. However, this phenomenon cannot be conclusively attributed to better ETV1 inhibition, as a fair comparison of the data is complicated by the fact that ERG and ETV1 are expressed in different cell-lines. Thus, the quality of response may also be a factor of differences between LNCaP and VCaP cells. Further experiments, such as measuring the magnitude of ERG and ETV1 response in the same cell-line, would be required to conclusively address this point.

Recent reports have suggested that ETS knock-down in prostate cancer cells may result in decreased proliferation in cells expressing these oncoproteins [21], [22]. Although the experiments in this manuscript were performed at doses and time intervals that were not toxic to the cells, there does appear to be a direct correlation between the expression of ETS proteins and YK-4-279 cytotoxicity. ETS-rearrangement negative PC-3 and DU-145 cells show minimal response to YK-4-279 treatment (IC50>100 µM). On the contrary, YK-4-279 is more toxic to both VCaP (IC50 = 9.55 µM after 72 h) and LNCaP cells (2.75 µM after 72 h). Hence, YK-4-279 can also be evaluated for its cytotoxic potentials in ETS-rearrangement positive prostate cancer cells in future studies.

The androgen dependent over-expression of ERG and ETV1 protein in prostate cancer cells has been directly implicated to increased invasion and metastasis. Furthermore, multiple studies have correlated the increased expression of these proteins with poor prognosis, higher Gleason scores and a lower incidence of recurrence free survival. Currently, androgen dependent signaling pathways in prostate cancer are targeted via castration and androgen receptor antagonists. The effects of these treatments can be in part attributed to the downregulation of rearranged ETS factors. Thus, the successful development of small molecule inhibitors of ERG and ETV1, such as YK-4-279, will represent a novel line of therapeutics aimed at preventing or treating metastatic disease, while saving patients the long-term effects of therapies targeting the androgen pathway.

## Materials and Methods

### Cell Culture

VCaP, LNCaP, PC-3 and DU-145 cells were obtained from ATCC (American Type Culture Collection, Manassas, VA). HUVECs were obtained from Lonza Biosciences (Allendale, NJ). VCaP cells were maintained in DMEM media supplemented with 10% Fetal Bovine Serum. LNCaP, PC-3 and DU-145 cells were maintained in RPMI media supplemented with 10% FBS and 1% HEPES. HUVEC cells were cultured in EBM-2 media (Lonza) supplemented with EGM-2 bullet kit (Lonza) containing growth factors, antibiotics and 5% FBS.

### Western-Blots

Protein lysates were prepared and western-blots performed as previously described [23]. ERG (sc-354), ETV1 (sc-1953) FLI1 (sc-356), PLAT (sc-5241) and actin (sc-1615) antibodies were purchased from Santa Cruz Biotechnology Inc. (Santa Cruz, CA). Anti-PLAU antibody was purchased from Calbiochem (Gibbstown, NJ).

### mRNA isolation and qPCR

mRNA was isolated using TRIzol (Invitrogen, Carlsbad, CA) and cDNA was prepared using transcriptor first-strand cDNA synthesis kit (Roche, San Francisco, CA) according to manufacturer's protocol. qRT-PCR was carried out using SYBR green (Roche) on a Mastercycler realplex^4^ instrument (Eppendorf, New York, NY). Gene expression was normalized to actin. Primer pairs are listed in Supplementary Table S1.

### Rearrangement status

Genomic DNA was isolated from PC-3, LNCaP and VCaP cells using Wizard genomic DNA extraction kit (Promega, Madison, WI) according to manufacturer's protocols. PCR was carried out using primers flanking rearrangement sites. Primer sequences can be found in Supplementary Table S1.

### Binding Kinetics

Steady state binding affinities were measured on a Biacore T100 instrument. Recombinant ERG and ETV1 (Origene, Rockville, MD) proteins were immobilized on CM5 chips by amine coupling and 6 different concentrations of YK-4-279 were injected over the surface in duplicates. SPR sensorgrams and K_D_ values were obtained using Biacore T100 software.

### Luciferase Assay

Cos-7 cells were co-transfected with a lentiviral plasmid expressing the most-commonly found truncated ERG mRNA, and a vector containing Id2 gene promoter driving expression of a luciferase gene. Transfection was carried out using Fugene 6 (Roche) according to the manufacturer's protocols. A lentiviral vector expressing LacZ was used as a negative control. Cells were allowed to express ERG for 48 hours and subsequently they were treated with 10 µM YK-4-279. Luciferase activity was measured after 24 h using a dual luciferase assay kit according the manufacturer's protocol (Promega, Madison, WI). Results were normalized to total protein concentration. Statistical analysis was performed using GraphPad Prism 4.0.

### Androgen and YK-4-279 treatment

For androgen treatment, cells were seeded in phenol-red free media containing 10% charcoal-stripped FBS and allowed to attach to the cell-culture dish overnight. Subsequently, cells were serum starved for 48 hours in phenol-red free media and then stimulated with 10 nM R1881 (Sigma, St-Louis, MO) for 2 days.

YK-4-279 was dissolved in DMSO to prepare 10 mM stock. Logarithmically growing cells were treated with 1 µM or 10 µM YK-4-279 for 48 hours prior to assessing for gene expression.

### siRNA ERG knockdown

Transient ERG knockdown was performed using a custom siRNA (5′-CGACATCCTTCTCTCACAT-3′) directed against the C-terminus of ERG (Dharmacon, Lafayette, CO) [21]. 50 nM siRNA was transfected using Lipofectamine 2000 (Invitrogen) according to the manufacturer's protocols. Cells were analyzed for ERG knockdown 5 days after transfection with siRNA.

### Transient ERG Expression

PC-3 cells were transfected with a pLenti6/V5-DEST plasmid (Invitrogen) expressing the most-commonly found truncated ERG isoform. Transfection was carried out using Fugene 6 reagent (Roche) according to the manufacturer's protocols for 48 hours.

### HUVEC Invasion

The anti-invasive potential of YK-4-279 was measured by using the technique of electric cell impedance sensing (ECIS) on ECIS Z instrument (Applied Biophysics, Troy, NY) and xCELLigence system (Roche). Briefly, 250,000 HUVEC cells were seeded in 8W10E+ arrays with electrode circuitry at well bottom to measure electrical resistance. Following formation of a confluent HUVEC monolayer (app. 21–24 hrs), the invading prostate cancer cells were added at a density of 100,000 cells per well in DMEM or RPMI media containing the indicated drug concentrations. Tumor cells were pre-treated for 24–48 hours with YK-4-279 before addition. This time point of tumor cell addition was accepted as 0 hr of treatment and invasion was monitored during the following 12 hours by measuring changes in resistance at the cell-electrode interphase. The experiments were performed in duplicates. Resistance was normalized to time of addition of invading cells.

### Scratch Assay

Cells were plated and allowed to form a confluent mono-layer. The cell-surface was scratched using a p-200 pipette tip. Cells were allowed to fill the scratched area and monitored over the course of 72 hours. Images were taken using a Nikon Eclipse Ti microscope (Nikon, Melville, NY). Cell motility was quantified by measuring the distance between the migrating cell boundaries.

### Chromatin Immunoprecipitation (ChIP)

PC-3 cells were transfected with a pLenti6/V5-DEST plasmid (Invitrogen) expressing the most commonly found truncated ERG isoform. Transfection was carried out using Fugene 6 reagent(Roche) according to the manufacturer's protocols for 24 hours. Cells were then treated for 6 hrs with 10 µM vehicle or YK-4-279. ChIP was carried out using EZMagna Protein A ChIP Kit from Millipore according to the manufacturer's instructions. Immunoprecipitation was carried out using 2 µg of ERG antibody (SC- 354x, Santa Cruz Biotechnology), 2 µg Normal Rabbit IgG (Sigma Aldrich) and 1 µg of Pol II (Millipore). PCR was carried out using primers previously published for positive ERG binding at the PLAU promoter in prostate cells [9]. A PCR profile of 94°C–5 min : 1 cycle, 94°C–30 sec, 55°C–30 sec, 72°C–1 min : 35 cycles, 72°C–5 min: 1 cycle was used on an Eppendorf Realplex4 thermocycler.

### Statistical Analysis

Groups were compared using a two-tailed Student's t-test (Prism 4, GraphPad Software, La Jolla, CA) and p<0.05 was considered significant.

## Supporting Information

Figure S1 **SPR sensorgrams for YK-4-279 binding to ERG and ETV1.** Steady state binding affinities were measured by injecting 6 different concentrations of YK-4-279 over recombinant ERG and ETV1 proteins immobilized on the surface of CM5 chips in a Biacore T100 instrument. SPR sensorgrams were obtained using Biacore T100 software.
(TIF)

Figure S2 **YK-4-279 is not a cytostatic agent.** VCaP (10,000 cells/well), LNCaP-high passage (10,000 cells/well), LNCaP-low passage (10,000 cells/well) and PC-3 (5,000 cells/well) cells were seeded overnight in xCELLigence E-16 plates and allowed to adhere to the well-bottom. The xCELLigence E-16 plates well-bottom is covered with miniature gold-electrodes which measure changes in electrical resistance on the surface of the electrodes. Changes in electrical resistance are represented as a dimensionless parameter termed cell-index, and is directly proportional to the area of well-bottom covered by electrodes. Approximately 20 hours after seeding prostate cancer cells, culture media was replaced with fresh media containing 1 µM (LNCaP, PC-3) or 10 µM (VCaP) YK-4-279. Cell proliferation was monitored over 72 hours.
(TIF)

Figure S3 **YK-4-279 inhibits LNCaP cell motility.** Cells were plated and allowed to form a confluent mono-layer. Cells were treated with 10 µg/ml mitomycin-C for 2 hours prior to scratch assay, as described previously [24], [25]. After mitomycin-C treatment, fresh media was added and the cell-surface was scratched using a p-200 pipette tip. Cells were allowed to fill the scratched area and monitored over the course of 60 hours. Cell motility was quantified by measuring the area of scratch not covered with migrating cells.. Motility was expressed relative to vehicle treated conditions. * ; p<0.0001
(TIF)

Figure S4 **ERG interacts with RHA in VCaP cells.** Yk-4-279 does not block the interaction between ERG and RHA. 9×10^7^ VCaP cells were seeded in 15 cm dishes and allowed to attach and spread for 48 hours. Cells were treated with 10 µM YK-4-279 for 24 h. Immunoprecipitation was performed as previously described [11].
(TIF)

Figure S5 **YK-4-279 does not inhibit ERG or ETV1 binding to DNA.** a) Recombinant ERG or ETV1 proteins were immobilized on the surface of a Biacore CM5 chip by amine coupling. Wild-type double-stranded oligonucleotides (ATGTAGACC**GGAA**GTAACTA) containing the consensus Ets binding site “GGAA” were injected in 5 µM triplicates over the surface of the chip in presence or absence of 50 µM YK-4-279. Binding of DNA to recombinant ERG or ETV1 was measured using Biacore T100 software. b) ChIP assay was performed by transfecting PC-3 cells with a lentiviral vector expressing ERG. Cells were treated for 6 hrs with 10 µM vehicle or YK-4-279. YK-4-279 did not inhibit binding of ERG to PLAU promoter.
(TIF)

Table S1 **Primers List**
(DOCX)
