# Supplementary figures and images for: YK-4-279 Inhibits ERG and ETV1 Mediated Prostate Cancer Cell Invasion
Source: PLoS One. 2011 Apr 29;6(4):e19343. doi: 10.1371/journal.pone.0019343 (PMC3084826; doi:10.1371/journal.pone.0019343)

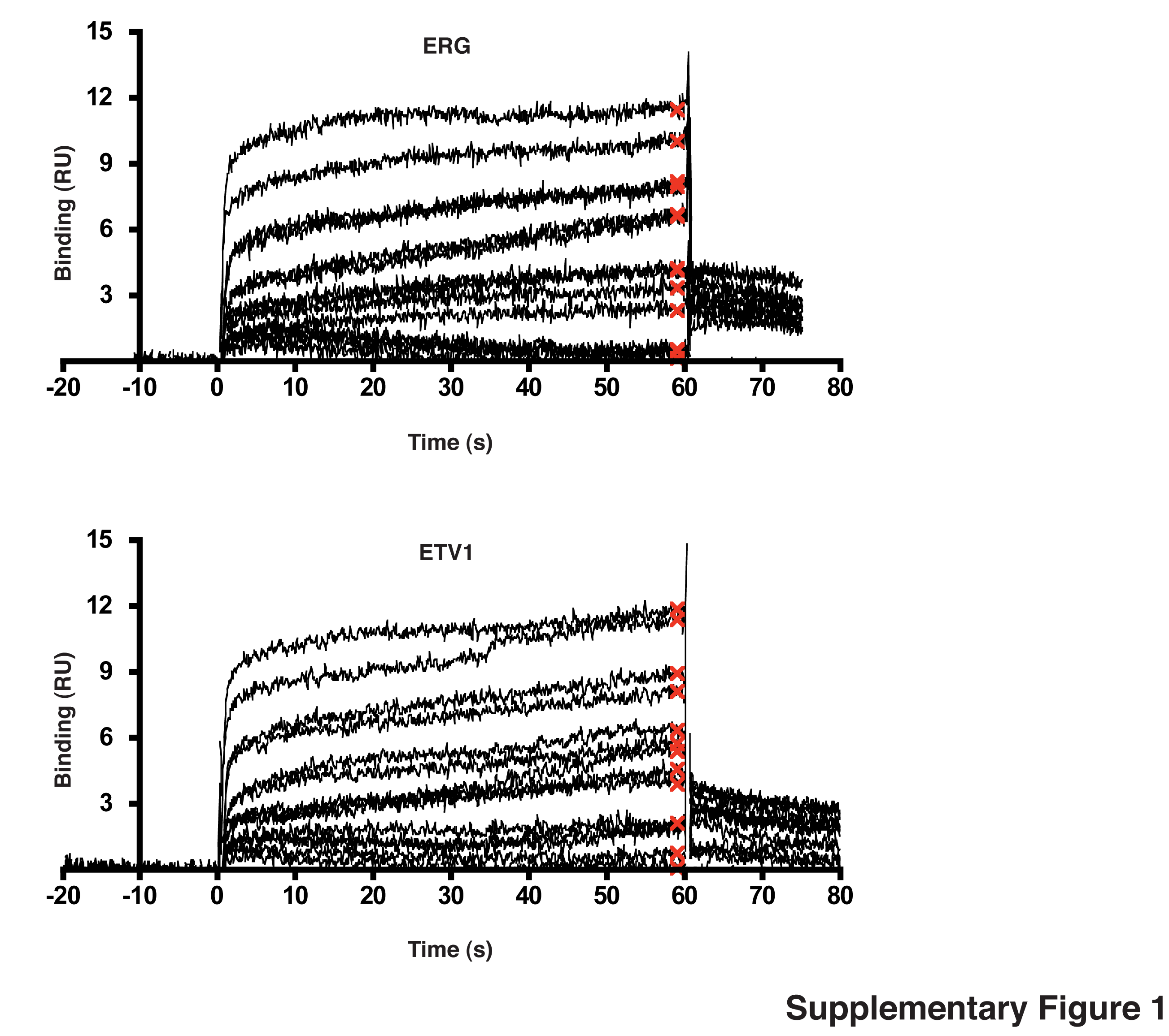

Supplement: Figure S1 — SPR sensorgrams for YK-4-279 binding to ERG and ETV1. Steady state binding affinities were measured by injecting 6 different concentrations of YK-4-279 over recombinant ERG and ETV1 proteins immobilized on the surface of CM5 chips in a Biacore T100 instrument. SPR sensorgrams were obtained using Biacore T100 software. (TIF) [file pone.0019343.s001.tif]

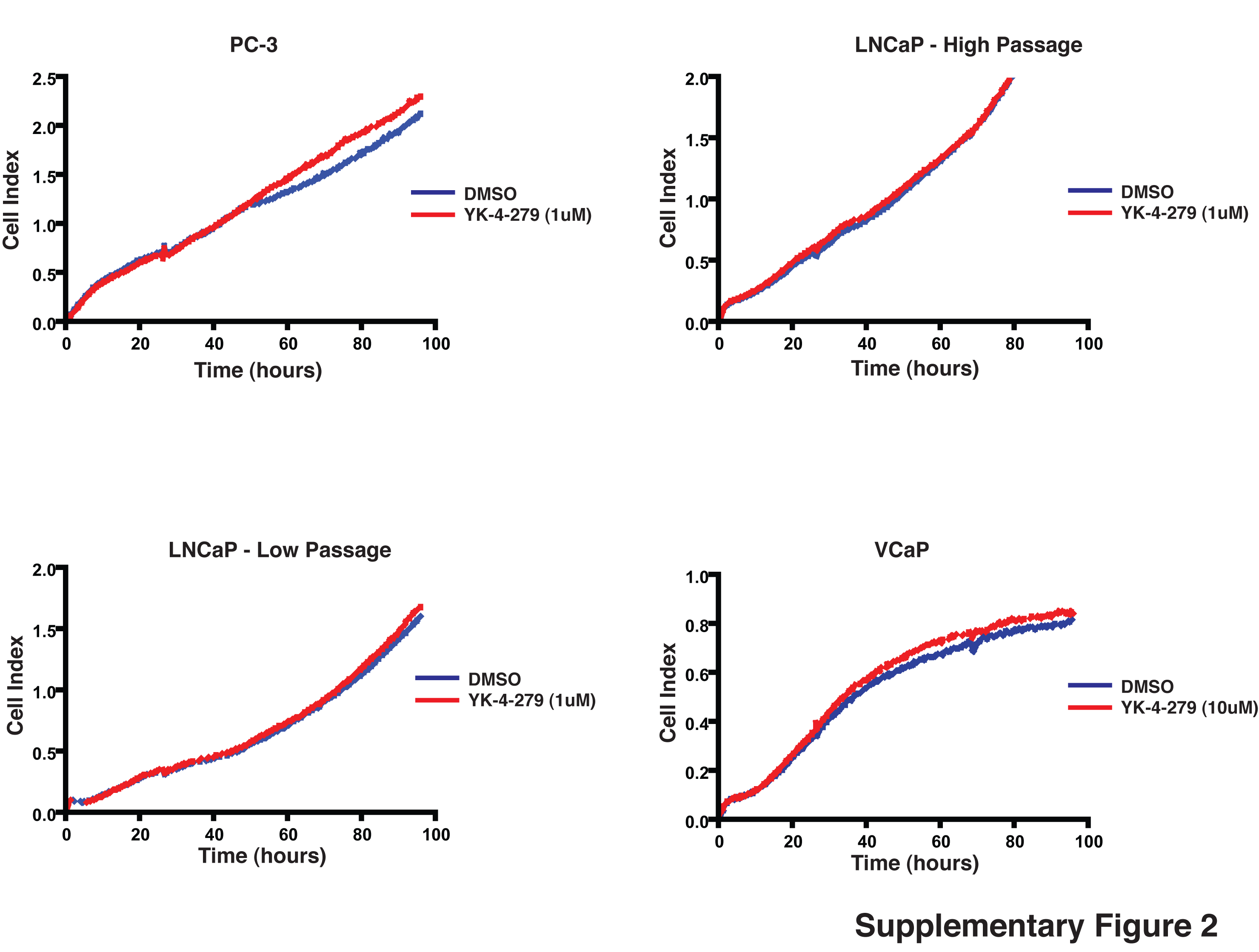

Supplement: Figure S2 — YK-4-279 is not a cytostatic agent. VCaP (10,000 cells/well), LNCaP-high passage (10,000 cells/well), LNCaP-low passage (10,000 cells/well) and PC-3 (5,000 cells/well) cells were seeded overnight in xCELLigence E-16 plates and allowed to adhere to the well-bottom. The xCELLigence E-16 plates well-bottom is covered with miniature gold-electrodes which measure changes in electrical resistance on the surface of the electrodes. Changes in electrical resistance are represented as a dimensionless parameter termed cell-index, and is directly proportional to the area of well-bottom covered by electrodes. Approximately 20 hours after seeding prostate cancer cells, culture media was replaced with fresh media containing 1 µM (LNCaP, PC-3) or 10 µM (VCaP) YK-4-279. Cell proliferation was monitored over 72 hours. (TIF) [file pone.0019343.s002.tif]

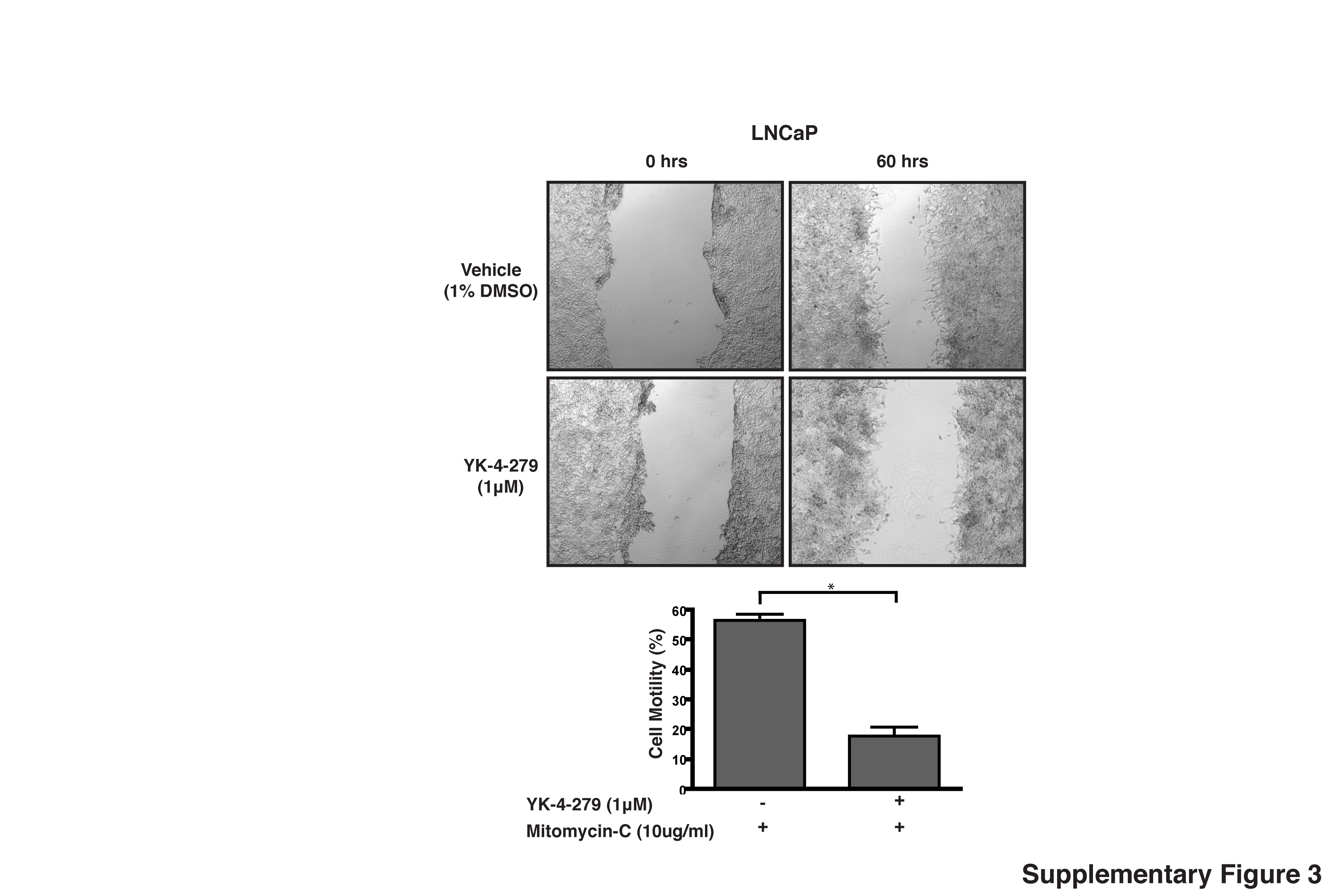

Supplement: Figure S3 — YK-4-279 inhibits LNCaP cell motility. Cells were plated and allowed to form a confluent mono-layer. Cells were treated with 10 µg/ml mitomycin-C for 2 hours prior to scratch assay, as described previously [24], [25]. After mitomycin-C treatment, fresh media was added and the cell-surface was scratched using a p-200 pipette tip. Cells were allowed to fill the scratched area and monitored over the course of 60 hours. Cell motility was quantified by measuring the area of scratch not covered with migrating cells.. Motility was expressed relative to vehicle treated conditions. * ; p<0.0001 (TIF) [file pone.0019343.s003.tif]

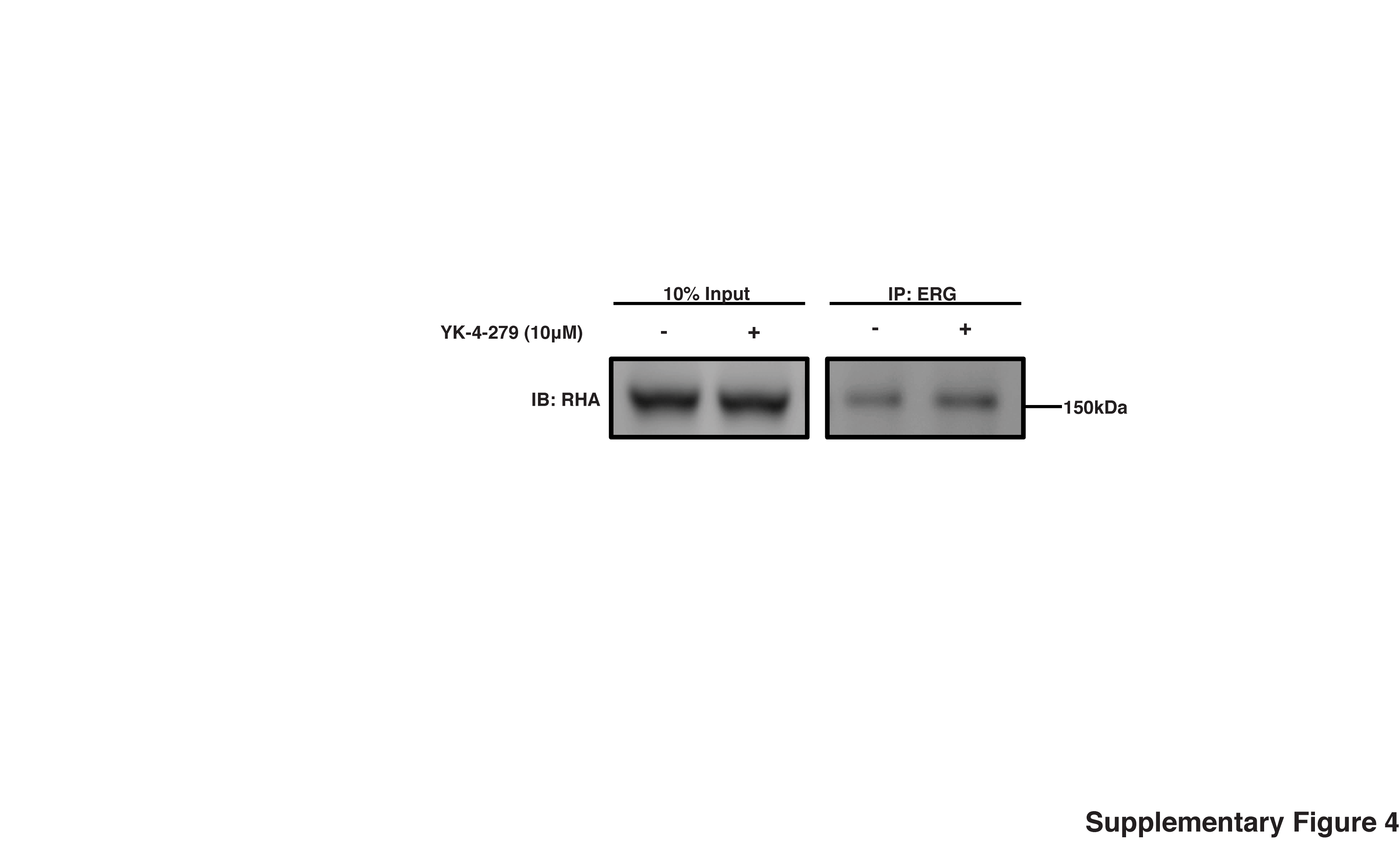

Supplement: Figure S4 — ERG interacts with RHA in VCaP cells. Yk-4-279 does not block the interaction between ERG and RHA. 9×10^7^ VCaP cells were seeded in 15 cm dishes and allowed to attach and spread for 48 hours. Cells were treated with 10 µM YK-4-279 for 24 h. Immunoprecipitation was performed as previously described [11]. (TIF) [file pone.0019343.s004.tif]

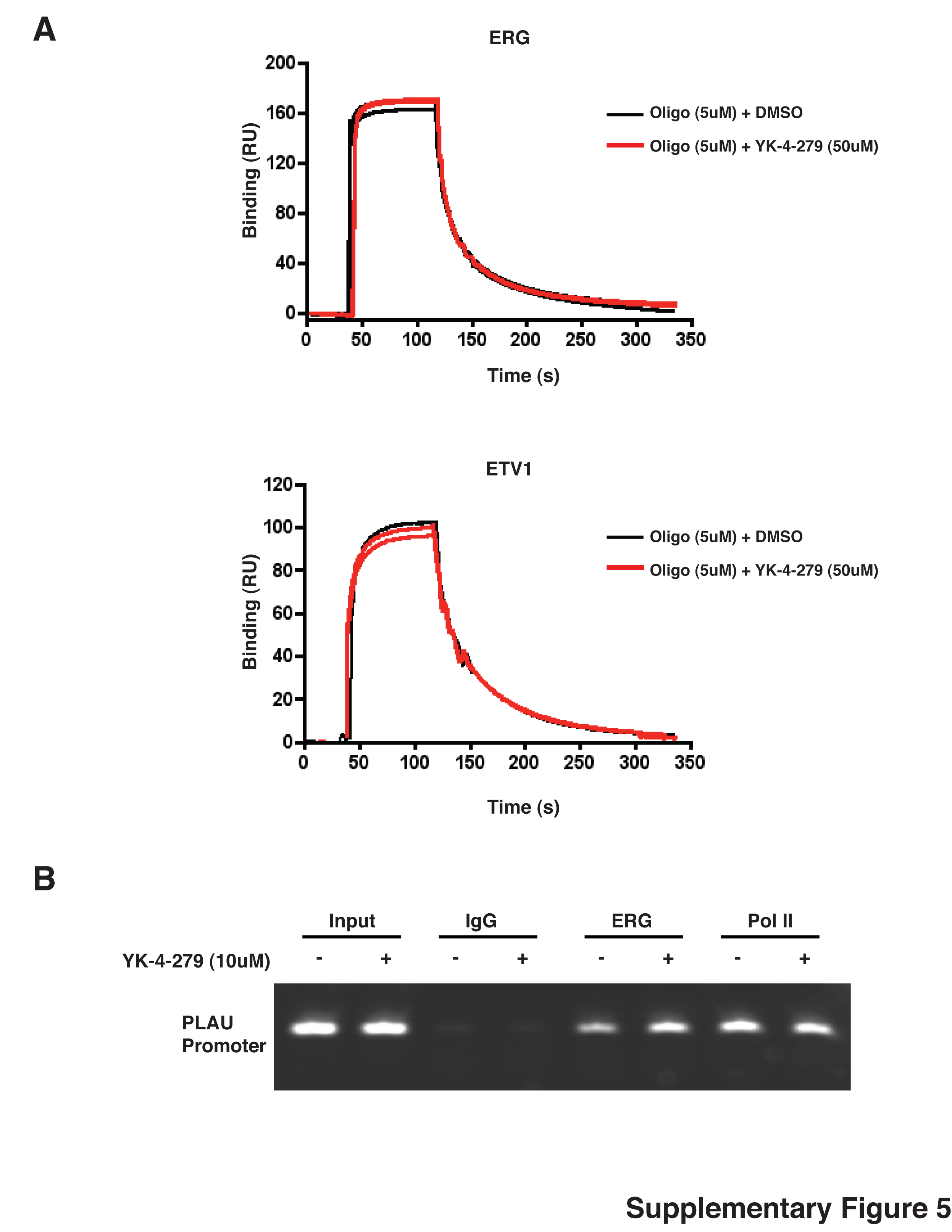

Supplement: Figure S5 — YK-4-279 does not inhibit ERG or ETV1 binding to DNA. a) Recombinant ERG or ETV1 proteins were immobilized on the surface of a Biacore CM5 chip by amine coupling. Wild-type double-stranded oligonucleotides (ATGTAGACCGGAAGTAACTA) containing the consensus Ets binding site “GGAA” were injected in 5 µM triplicates over the surface of the chip in presence or absence of 50 µM YK-4-279. Binding of DNA to recombinant ERG or ETV1 was measured using Biacore T100 software. b) ChIP assay was performed by transfecting PC-3 cells with a lentiviral vector expressing ERG. Cells were treated for 6 hrs with 10 µM vehicle or YK-4-279. YK-4-279 did not inhibit binding of ERG to PLAU promoter. (TIF) [file pone.0019343.s005.tif]
