# Supplementary material for: YK-4-279 Inhibits ERG and ETV1 Mediated Prostate Cancer Cell Invasion
Source: PLoS One. 2011 Apr 29;6(4):e19343. doi: 10.1371/journal.pone.0019343 (PMC3084826; doi:10.1371/journal.pone.0019343)
Supplement: Table S1 — Primers List (DOCX) [file pone.0019343.s006.docx]

**Supplementary Table 1: Primers List**

| ERG-f | CGC AGA TTA TCG TGC CAG CAG AT |
| --- | --- |
| ERG-r | CCA TAT TCT TTC ACC GCC CAC TCC |
| ETV1-f | TAC CCC ATG GAC CAC AGA TT |
| ETV1-r | CAC TGG GTC GTG GTA CTC CT |
| PLAU-f | TAC GGC TCT GAA GTC ACC ACC AAA A |
| PLAU-r | CCC CAG CTC ACA ATT CCA GTC AA |
| ADAM19-f | GCC TAT GCC CCC TGA GAG TG |
| ADAM19-r | GCT TGA GTT GGC CTA GTT TGT TGT TC |
| PLAT-f | CAC TGG GCC TGG GCA AAC ATA |
| PLAT-r | CAC GTC AGC CTG CGG TTC TTC |
| MMP13-f | TTG AGC TGG ACT CAT TGT CG |
| MMP13-r | GGA GCC TCT CAG TCA TGG AG |
| PSA-f | TTG TGG CCT CTC GTG GCA GGG CAG T |
| PSA-r | TGG TCA CCT TCT GAG GGT GAA CTT GC |
| ERG-rearrangement-1 | TAG GCG CGA GCT AAG CAG GAG |
| ERG-rearrangement-2 | GTA GGC ACA CTC AAA CAA CGA CTG G |
| ETV1-rearrangement-1 | CGC GAG CTA AGC AGG AGG C |
| ETV1-rearrangement-2 | CAG GCC ATG AAA AGC CAA ACT T |
